# Sleeping It Off: Sleep Quality Moderates the Association Between Family Bereavement and Heart Rate Variability

**DOI:** 10.1093/geroni/igab046.430

**Published:** 2021-12-17

**Authors:** Hye Won Chai, Dylan Jester, Soomi Lee, Susanna Joo, Debra Umberson, David Almeida

**Affiliations:** 1 The University of Texas at Austin, Austin, Texas, United States; 2 University of California San Diego, La Jolla, California, United States; 3 University of South Florida, Tampa, Florida, United States; 4 Yonsei University, Seodaemun-gu, Seoul-t'ukpyolsi, Republic of Korea; 5 Pennsylvania State University, University Park, Pennsylvania, United States

## Abstract

While previous studies evince a strong link between family bereavement and worse cardiovascular functioning, factors that may influence the association remain unexplored. This study examined the relation between experiencing the death of an immediate family member and heart rate variability (HRV) and whether the associations differed by sleep quality. The sample included respondents from the Midlife in the United States (MIDUS) Biomarker Project who reported losing an immediate family member – parents, spouse, siblings, or children – within a year before project (n = 94) and those who did not experience any deaths (n = 872). Results showed that the death of a family member was associated with worse HRV only among those who reported having a poor sleep quality and not for those with good sleep quality. These findings suggest that poor sleep quality may indicate psychophysiological vulnerability for those who experienced the death of an immediate family member.

